# Coronary artery calcium among patients with heterozygous familial hypercholesterolaemia

**DOI:** 10.1093/ehjopen/oead046

**Published:** 2023-05-03

**Authors:** Hayato Tada, Nobuko Kojima, Kan Yamagami, Akihiro Nomura, Atsushi Nohara, Soichiro Usui, Kenji Sakata, Kenshi Hayashi, Noboru Fujino, Masayuki Takamura, Masa-aki Kawashiri

**Affiliations:** Department of Cardiovascular Medicine, Graduate School of Medical Sciences, Kanazawa University, 13-1 Takara-machi, Kanazawa, 920-8641, Japan; Department of Cardiovascular Medicine, Graduate School of Medical Sciences, Kanazawa University, 13-1 Takara-machi, Kanazawa, 920-8641, Japan; Department of Cardiovascular Medicine, Graduate School of Medical Sciences, Kanazawa University, 13-1 Takara-machi, Kanazawa, 920-8641, Japan; Department of Cardiovascular Medicine, Graduate School of Medical Sciences, Kanazawa University, 13-1 Takara-machi, Kanazawa, 920-8641, Japan; Department of Clinical Genetics, Ishikawa Prefectural Central Hospital, Kanazawa, Japan; Department of Cardiovascular Medicine, Graduate School of Medical Sciences, Kanazawa University, 13-1 Takara-machi, Kanazawa, 920-8641, Japan; Department of Cardiovascular Medicine, Graduate School of Medical Sciences, Kanazawa University, 13-1 Takara-machi, Kanazawa, 920-8641, Japan; Department of Cardiovascular Medicine, Graduate School of Medical Sciences, Kanazawa University, 13-1 Takara-machi, Kanazawa, 920-8641, Japan; Department of Cardiovascular Medicine, Graduate School of Medical Sciences, Kanazawa University, 13-1 Takara-machi, Kanazawa, 920-8641, Japan; Department of Cardiovascular Medicine, Graduate School of Medical Sciences, Kanazawa University, 13-1 Takara-machi, Kanazawa, 920-8641, Japan; Department of Internal Medicine, Kaga Medical Center, Kaga, Japan

**Keywords:** Familial hypercholesterolaemia, LDL cholesterol, Genetics, LDL receptor, Coronary calcium score

## Abstract

**Aims:**

We aimed to determine if coronary artery calcium (CAC) is associated with cardiovascular disease (CVD) events, defined as CVD-related death, unstable angina, myocardial infarction, or staged revascularization among patients with heterozygous familial hypercholesterolaemia (HeFH) under primary prevention settings.

**Methods and results:**

Data of patients with FH admitted to Kanazawa University Hospital between 2000 and 2020, who underwent CAC measurement and were followed up (*n* = 622, male = 306, mean age = 54 years), were retrospectively reviewed. Risk factors for CVD events were determined using the Cox proportional hazard model. The median follow-up duration was 13.2 years (interquartile range: 9.8–18.4 years). We observed 132 CVD events during the follow-up period. The event rate per 1000 person-years for CAC scores of 0 [*n* = 283 (45.5%)], 1–100 [*n* = 260 (41.8%)], and >100 [*n* = 79 (12.7%)] was 1.2, 17.0, and 78.8, respectively. Log (CAC score + 1) was a significant predictor of the occurrence of CVD events (hazard ratio: 3.24; 95% confidence interval: 1.68–4.80; *P* < 0.0001) in the multivariate Cox regression analysis, independent of other factors. The risk discrimination of CVD events was enhanced by adding CAC information to other conventional risk factors (*C*-statistics: 0.833–0.934; *P* < 0.0001).

**Conclusion:**

The CAC score helps in further risk stratification in patients with HeFH.

## Structured Graphical Abstract

**Graphical Abstract:**
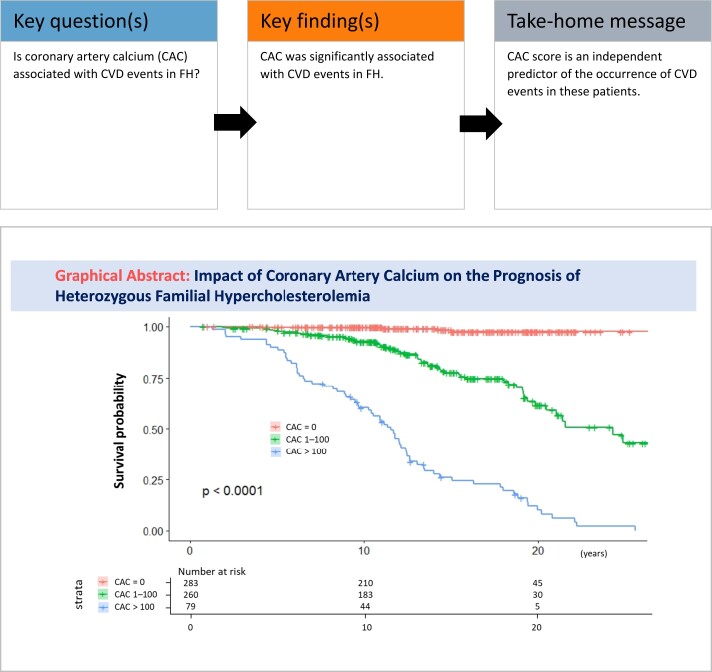

## Introduction

Patients with familial hypercholesterolaemia (FH) caused by pathogenic mutations in the low-density lipoprotein receptor (*LDLR*) or its associated genes, including apolipoprotein B (*APOB*), proprotein convertase subtilisin/kexin type 9 (*PCSK9*), and *LDLR* adaptor protein 1 (*LDLRAP1*), have an extremely high risk of atherosclerotic cardiovascular diseases (ASCVDs) caused by chronic exposure to high LDL cholesterol.^1–3^ Although their risk of ASCVDs is extremely high, studies have shown that the individual risk is heterogeneous.^4^ We and others have shown that many factors affect the actual individual risk of ASCVDs in patients with FH, such as age, sex, diabetes, hypertension, smoking, the presence of pathogenic variants, high-density lipoprotein (HDL) cholesterol, LDL cholesterol, and plaque load in the coronary arteries as measured by coronary computed tomography (CT).^5–9^ These factors, except for the presence of pathogenic variants of FH, are also associated with ASCVDs in individuals without FH.^10^ In contrast, recent studies have suggested that the presence and extent of coronary artery calcium (CAC), which is a surrogate of atherosclerotic plaque burden, are useful biomarkers for risk stratification.^11,12^ Notably, the absence of CAC (CAC score of 0) is favourable in general primary prevention settings.^13^ So, CAC has been shown not only as a good surrogate marker for the development of atherosclerosis but also as a good biomarker for risk stratification of cardiovascular disease (CVD) events among the general population (non-FH patients).^14^ However, data on the effects of CAC on the FH phenotype are limited.^15,16^ We aimed to determine if CAC is associated with CVD events, defined as CVD-related death, unstable angina, myocardial infarction, or staged revascularization among patients with heterozygous FH (HeFH) under primary prevention settings.

## Methods

### Study population

We analysed data from 932 patients diagnosed with HeFH using the Japan Atherosclerosis Society (JAS) 2017 criteria.^17^ These patients were admitted to Kanazawa University Hospital between 2000 and 2020 and underwent coronary CT. One hundred and twenty-one patients were excluded due to any history of coronary revascularization, 96 were excluded because of missing data, and 93 were excluded because they were lost to follow-up. Finally, in this study, 622 patients were included (see Supplementary material online, *Figure S1*).

### Clinical data assessment

Hypertension was defined as systolic blood pressure ≥ 140 mmHg, diastolic blood pressure ≥ 90 mmHg, or antihypertensive medication use. We adopted the Japan Diabetes Society’s definition of diabetes.^18^ The current smoking status of the patients was considered. Using automated instrumentation, we enzymatically measured the serum concentrations of triglycerides, HDL cholesterol, and total cholesterol. The LDL cholesterol level was enzymatically determined whether the triglyceride level was ≥400 mg/dL and using the Friedewald formula otherwise. A CVD event was defined as CVD-related death, unstable angina, myocardial infarction, or staged revascularization.

### Assessment of coronary artery calcium

Coronary CT was performed using a dual-source 64-slice system (Somatom Definition Flash; Siemens Medical Systems, Erlangen, Germany). The details were described in a previous study.^19^ The CAC score was assessed using the Agatston method using dedicated software (SYNAPSE VINCENT; Fujifilm Medical, Tokyo, Japan). When determining the precise location of calcified lesions was difficult, we referred to contrast-enhanced scans.

### Genetic analysis

We used a next-generation sequencer to evaluate genotypes. In brief, the coding regions of *APOB*, *LDLR*, *LDLRAP1*, and *PCSK9* were sequenced, as described previously.^20^ Copy number variations at the *LDLR* were also assessed, as described previously, using the eXome Hidden Markov Model.^21^ We used the standard American College of Medical Genetics and Genomics criteria (‘pathogenic’ or ‘likely pathogenic’) to determine whether the genetic variants were pathogenic.^22^

### Ethical considerations

The Ethics Committee of Kanazawa University approved this study (2015–219). All procedures met the ethical standards of the Human Research Committee (institutional and national) and the Declaration of Helsinki (1975, revised in 2008). All study participants provided informed consent for genetic analysis.

### Statistical analysis

Normally distributed continuous variables are presented as means ± standard deviations. Meanwhile, continuous variables that did not follow a normal distribution are presented as medians and interquartile ranges (IQRs). All comparisons between categorical variables were performed using Fisher’s exact test or the *χ*^2^ test, and the results are reported as numbers or percentages. For independent variables, Student’s *t*-test was used to compare the means of continuous variables, and the non-parametric Wilcoxon–Mann–Whitney rank sum test was used to compare the median values. For categorical variables, we performed the *χ*^2^ test or Fisher’s *post hoc* test as indicated. A linear regression model was used to analyse correlations between the LDL cholesterol year score and CAC. The significance of trends was assessed using the Cochran–Armitage trend test or Jonckheere–Terpstra trend test. The correlations between these variables were evaluated using the Cox proportional hazard model. The Fine–Gray model was also used to estimate the hazard ratio (HR) for CVD events. Beginning at baseline, cumulative Kaplan–Meier survival curves were generated to compare the times to the first CVD events. The predictive performance of the variables under consideration was estimated using receiver operating characteristic (ROC) analysis and *C*-statistics. DeLong *et al.*’s^23^ method was used to compare the *C*-statistic estimates. In addition, we calculated net reclassification improvement (NRI) and integrated discrimination improvement (IDI) by adding the information of CAC scores on top of classical risk factors. R (https://www.r-project.org) was used for all statistical analyses. Additionally, for each CAC stratum, the CVD events per 1000 person-years were calculated. *P*-values of less than 0.05 were used to denote statistical significance.

## Results

### Clinical characteristics

The study participants’ clinical characteristics are presented in *Table 1*. The patients’ mean age was 54 years, and ∼50% of them were male. At baseline, the median LDL cholesterol level was 229 mg/dL. Overall, 425 patients (68.3%) had a pathogenic variant of FH. We found 397 patients with a pathogenic variant in *LDLR* and 28 patients with a pathogenic variant in *PCSK9*. There was no patient with an *APOB* or *LDLRAP1* variant. When we divided the patients according to their CAC score, we found significant trends in variables, such as age, sex, diabetes, hypertension, smoking, total cholesterol, triglycerides, HDL cholesterol, baseline LDL cholesterol, and the LDL cholesterol year score. When we divided the patients into two groups based on the occurrence of CVD events, we observed several differences in variables, such as age, sex, diabetes, hypertension, smoking, total cholesterol, HDL cholesterol, baseline LDL cholesterol, the LDL cholesterol year score, the prevalence of FH pathogenic variants, and the CAC score between the groups (see Supplementary material online, *Table S1*). A summary of the follow-up medical treatments administered is presented in Supplementary material online, *Table S2*. Most patients received statin therapy, followed by ezetimibe and colestimide. We identified 83 pathogenic variants in 425 patients (see Supplementary material online, *Table S3*).

**Table 1:** Baseline characteristics

Variables	All	CAC = 0	CAC 1−100	CAC > 100	*P*-value for trend
	*n* = 622	*n* = 283	*n* = 260	*n* = 79	
Age (years)	54 ± 13	47 ± 12	59 ± 11	63 ± 12	<0.0001
Male (%)	306 (49.2%)	109 (38.5%)	148 (56.9%)	49 (62.0%)	<0.0001
Hypertension (%)	200 (32.1%)	46 (16.3%)	104 (40.0%)	50 (63.3%)	<0.0001
Diabetes (%)	64 (10.3%)	14 (4.9%)	36 (13.8%)	14 (17.7%)	<0.0001
Smoking (%)	217 (34.9%)	67 (23.7%)	110 (42.3%)	40 (50.6%)	<0.0001
Total cholesterol (mg/dL)	318 (286–360)	319 (288–362)	314 (286–346)	321 (285–393)	0.034
Triglyceride (mg/dL)	130 (91–176)	124 (78–167)	134 (103–176)	145 (86–187)	<0.0001
HDL cholesterol (mg/dL)	46 (39–56)	50 (41–61)	45 (38–55)	43 (34–53)	<0.0001
LDL cholesterol (at baseline, mg/dL)	229 (205–275)	227 (204–277)	228 (215–261)	259 (212–310)	<0.0001
LDL cholesterol (at follow-up, mg/dL)	108 (90–127)	110 (93–130)	104 (88–123)	109 (89–130)	0.24
LDL cholesterol year score (years × mg/dL)	12692 (10085–15689)	11118 (9051–13387)	13860 (11288–16074)	16195 (13333–19899)	<0.0001
FH pathogenic variants (%)	425 (68.3%)	193 (68.2%)	164 (63.1%)	68 (86.1%)	0.14

FH, familial hypercholesterolaemia; CAC, coronary artery calcium.

### Coronary artery calcium distribution according to the occurrence of cardiovascular disease events

**Figure 1:**
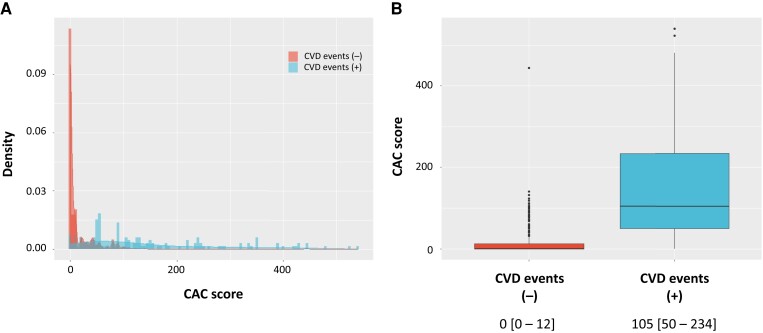
Coronary artery calcium distribution according to the incidence of cardiovascular disease events. (*A*) Histograms with density. Red indicates patients without cardiovascular disease events. Blue indicates patients with cardiovascular disease events. (*B*) Boxplots. Red indicates patients without cardiovascular disease events. Blue indicates patients with cardiovascular disease events. CAC, coronary artery calcium; CVD, cardiovascular disease.

The overall CAC distribution was highly skewed to the right (*Figure 1A*). When we examined CAC according to the occurrence of CVD events, it was significantly higher in patients with CVD events than in patients without CVD events [105 (50–234) vs. 0 (0–12); *P* < 0.0001] (*Figure 1B*).

### Correlation between the low-density lipoprotein cholesterol year score and coronary artery calcium

**Figure 2:**
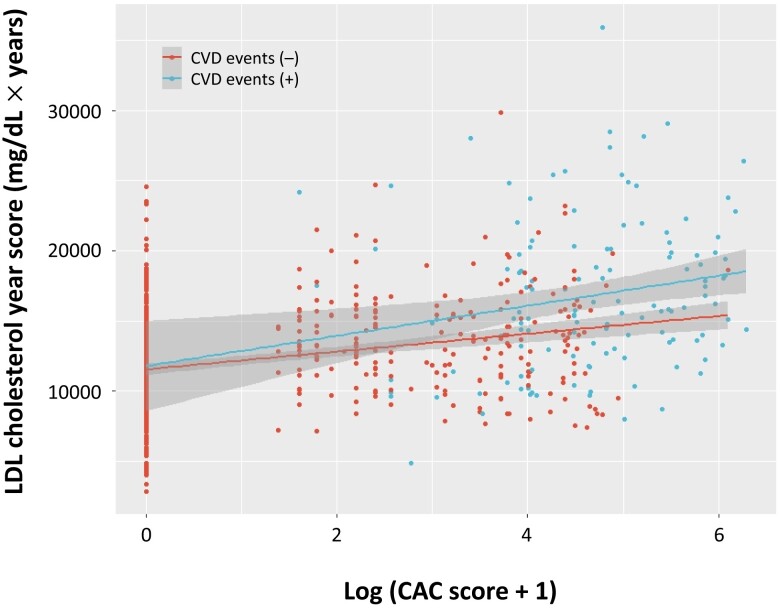
Correlation between the low-density lipoprotein cholesterol year score and coronary artery calcium. Red indicates patients without cardiovascular disease events. Blue indicates patients with cardiovascular disease events. The *X*-axis represents log (coronary artery calcium score + 1). The *Y*-axis represents the low-density lipoprotein cholesterol year score. CAC, coronary artery calcium; CVD, cardiovascular disease; LDL, low-density lipoprotein.

We hypothesized that the accumulation of LDL cholesterol over the years caused the development of CAC in patients with FH. We found that the LDL cholesterol year score, which represents the exposure to lifelong high LDL cholesterol, was significantly correlated with CAC in patients with and without CVD events (Spearman’s *r* = 0.29; *P* < 0.0001 and Spearman’s *r* = 0.26; *P* = 0.003, respectively) (*Figure 2*).

### Factors associated with cardiovascular disease events

Overall, 132 patients had CVD events (i.e. CVD-associated death, unstable angina, myocardial infarction, and staged revascularization) over a median follow-up period of 13.2 years (IQR, 9.8–18.4 years) (see Supplementary material online, *Table S4*). We evaluated the risk factors for CVD events using the Cox proportional hazard model and found that age [HR = 1.06; 95% confidence interval (CI) = 1.04–1.08; *P* < 0.0001], male sex (HR = 1.60; 95% CI = 1.05–2.15; *P* = 0.009), hypertension (HR = 2.58; 95% CI = 1.80–3.36; *P* < 0.0001), diabetes (HR = 2.10; 95% CI = 1.20–3.00; *P* = 0.0001), smoking (HR = 2.88; 95% CI = 1.86–3.90; *P* = 0.0001), LDL cholesterol (HR = 1.01; 95% CI = 1.00–1.02; *P* = 0.023, per 10 mg/dL), and the presence of pathogenic variants (HR = 3.18; 95% CI = 2.00–4.36; *P* < 0.0001) were significantly associated with CVD events (*Table 2*). In addition to these classical risk factors, the CAC score [log (CAC + 1)] was also associated with CVD events (HR = 3.24; 95% CI = 1.68–4.80; *P* < 0.0001). The Fine–Gray regression model was also used to estimate the HR for CVD events, considering death as a competing risk event, and we found almost the same results (see Supplementary material online, *Table S5*).

**Table 2:** Factors associated with CVD events

Variable	HR	95% CI	*P*-value
Age (per year)	1.06	1.04–1.08	<0.0001
Male (yes vs. no)	1.60	1.05–2.15	0.009
Hypertension (yes vs. no)	2.58	1.80–3.36	<0.0001
Diabetes (yes vs. no)	2.10	1.20–3.00	0.0001
Smoking (yes vs. no)	2.88	1.86–3.90	0.0001
LDL cholesterol (per 10 mg/dL)	1.01	1.00–1.02	0.023
Pathogenic variants (vs. without variants)	3.18	2.00–4.36	<0.0001
Log (CAC + 1)	3.24	1.68–4.80	<0.0001

HR, hazard ratio; CI, confidence interval; CAC, coronary artery calcium; CVD, cardiovascular event.

### Risk discrimination by coronary artery calcium

**Figure 3:**
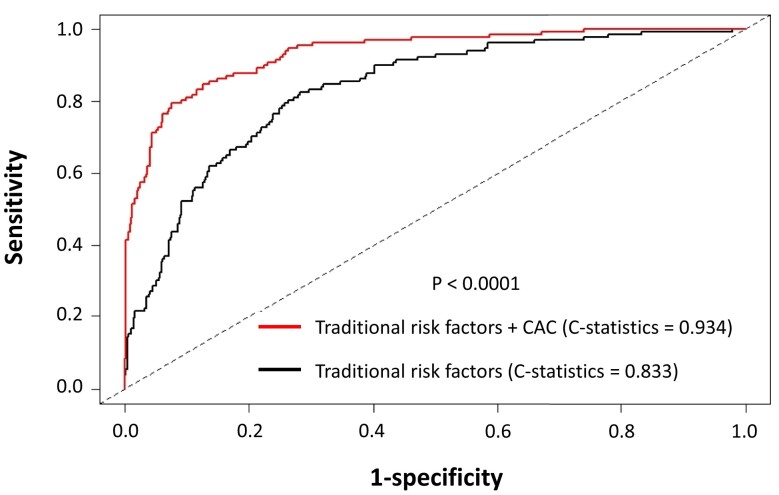
Receiver operating characteristic analysis predicting the incidence of cardiovascular disease events. Black line indicates the receiver operating characteristic curve using traditional risk factors (i.e. age, sex, smoking, diabetes, hypertension, and low-density lipoprotein cholesterol). Red curve indicates the receiver operating characteristic curve using traditional risk factors (i.e. age, sex, smoking, diabetes, hypertension, and low-density lipoprotein cholesterol) and coronary artery calcium. The *X*-axis represents specificity. The *Y*-axis represents sensitivity. CAC, coronary artery calcium; LDL, low-density lipoprotein.

We investigated whether the risk discrimination of a model that included CAC differed significantly from that of a model based only on traditional risk factors, such as age, sex, smoking, diabetes, hypertension, and LDL cholesterol. The *C*-statistic for the model based only on traditional risk factors was 0.833 (95% CI = 0.802–0.865) and increased to 0.934 (95% CI = 0.912–0.956) (*P* < 0.0001) after incorporating CAC into the model (*Figure 3*). In addition, we found significant improvements in risk discrimination for CVD events by adding CAC score information on top of classical risk factors, including age, sex, smoking, diabetes, hypertension, and LDL cholesterol (continuous NRI = 0.17, 95% CI = 0.09–0.26, *P* < 0.0001; IDI = 0.04, 95% CI = 0.032–0.048, *P* < 0.0001)

### Prognosis according to coronary artery calcium strata

**Figure 4:**
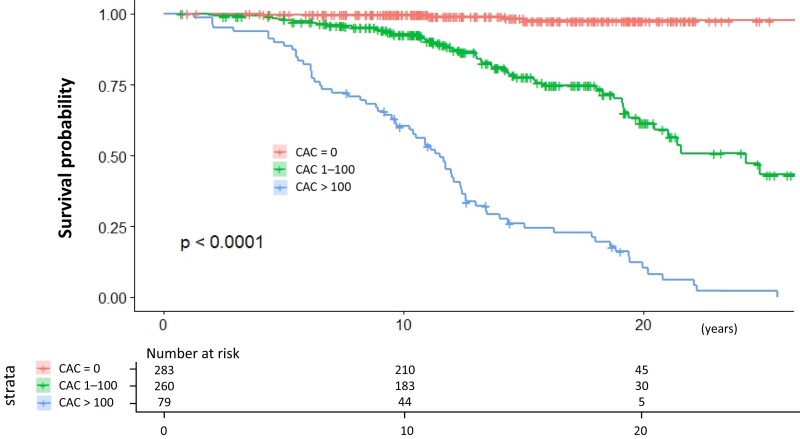
Kaplan–Meier survival curves. Red indicates patients with a coronary artery calcium score of 0. Green indicates patients with coronary artery calcium scores of 1–100. Blue indicates patients with coronary artery calcium scores of >100. CAC, coronary artery calcium.

Our assessment of the survival curve according to CAC strata revealed that patients with CAC scores of 1–100 had worse outcomes than patients with a CAC score of 0, and patients with CAC scores of >100 had the worst outcome among the three groups (*Figure 4*). The event rate per 1000 person-years for CAC scores of 0 [*n* = 283 (45.5%)], 1–100 [*n* = 260 (41.8%)], and >100 [*n* = 79 (12.7%)] was 1.2, 17.0, and 78.8, respectively.

### Impact of low-density lipoprotein cholesterol treatment target attainment

When we divided the patients into two groups based on the attainment of LDL cholesterol treatment targets, we found that patients who attained treatment targets (LDL cholesterol < 100 mg/dL) had better prognoses (see Supplementary material online, *Figure S2*).

## Discussion

In this study, we investigated the prognostic impact of CAC on the occurrence of CVD events in patients with HeFH under primary prevention settings. We found that CAC was significantly associated with CVD events, leading to incremental improvement in risk stratification in patients with HeFH.

A growing body of evidence suggests that patients with FH have an extremely elevated risk of ASCVDs.^24^ However, great diversity still exists regarding the severity of phenotypes among these ‘monogenic’ disorders. Many factors, including traditional risk factors, such as hypertension, diabetes, and smoking, affect their phenotype. In addition to such classical risk factors, several emerging factors, including biomarkers (e.g. remnant cholesterol), imaging (coronary plaque burden), and genetics (pathogenic variants of FH), are useful in further risk stratification.^25,26^ Note that these traditional and emerging risk factors have been useful for risk stratification in the general population. Among these emerging factors, CAC has been attracting much attention because of its predictive power for future CVD events, particularly its negative predictive power, where almost all patients with a CAC score of 0 have been free from CVD events in the general population.^27^ However, only a few studies have specifically investigated this factor among patients with FH in a super high-risk group.^15,16^ In this study, we have clarified that a CAC score of 0 is also an important negative biomarker of the occurrence of CVD events in patients with HeFH.

This study has several limitations. First, this was a single-centre retrospective study. Therefore, our study findings may not apply to other patients. However, our institution has one of Japan’s largest databases and a long history of treating patients with FH. Furthermore, we believe that this is the first study focusing on when the development of CAC progression starts in patients with HeFH. Further studies investigating European and other ethnicities should be able to refer to our results, given that most of the physicians are interested in differences among races. Second, we were unable to account for treatment discontinuations or alterations during follow-up, which may have affected the study findings. Third, some patients were excluded from the analysis due to missing data or because they were lost to follow-up, which could have impacted the study findings. Fourth, we estimated the correlation coefficient under the assumption of linear regression, which may not apply to the development of CAC. Fifth, there is no comparable ‘control group’ in this study, which may limit the interpretation of our results. In this regard, there is a study suggesting that CAC rarely develops in Asians before the age of 40 in men and before the age of 50 in women^28^ in contrast to Black and White populations.^29^ We believe that vascular ageing in patients with HeFH estimated from the development of CAC was much more advanced compared with that in the general population. Sixth, we could not assess the stroke events in this study. Further studies comprehensively evaluating these conditions will be useful in estimating their overall risk assessments.

## Conclusions

In conclusion, the results showed that the CAC score is an independent predictor of the occurrence of CVD events in these patients. This helps in further risk stratification for aggressive treatments.

## Lead author biography


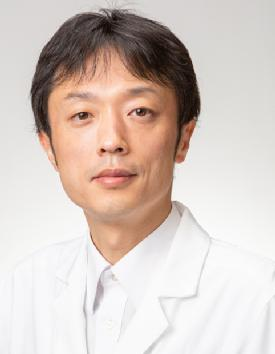
 **Hayato Tada MD, PhD**

**Table:** Experiences:

2003–04	Resident in Medicine, Kanazawa University Hospital, Kanazawa, Japan
2004–05	Clinical Fellow in Cardiology and Internal Medicine, Kouseiren Takaoka Hospital, Takaoka, Japan
2005–06	Clinical Fellow in Fukui Cardiovascular Center, Fukui, Japan
2006–11	Medical Staff in Division of Cardiovascular Medicine, Kanazawa University Hospital, Kanazawa, Japan
2012–14	Research Scholar in Massachusetts General Hospital, Center for Human Genetic Research, Boston, MA, USA
2014–	Assistant Professor in Department of Cardiovascular Medicine, Graduate School of Medical Sciences, Kanazawa University, Kanazawa, Japan

## Supplementary Material

oead046_Supplementary_Data

## Data Availability

Requests to access the datasets should be directed to H.T.
