# Supplementary material for: Coronary artery calcium among patients with heterozygous familial hypercholesterolaemia
Source: Eur Heart J Open. 2023 May 3;3(3):oead046. doi: 10.1093/ehjopen/oead046 (PMC10182732; doi:10.1093/ehjopen/oead046)
Supplement: oead046_Supplementary_Data [file oead046_supplementary_data.docx]

Supplementary Material

**Supplemental Figure 1. Strengthening the Reporting of Observational Studies in Epidemiology flow diagram for study inclusion.**

**
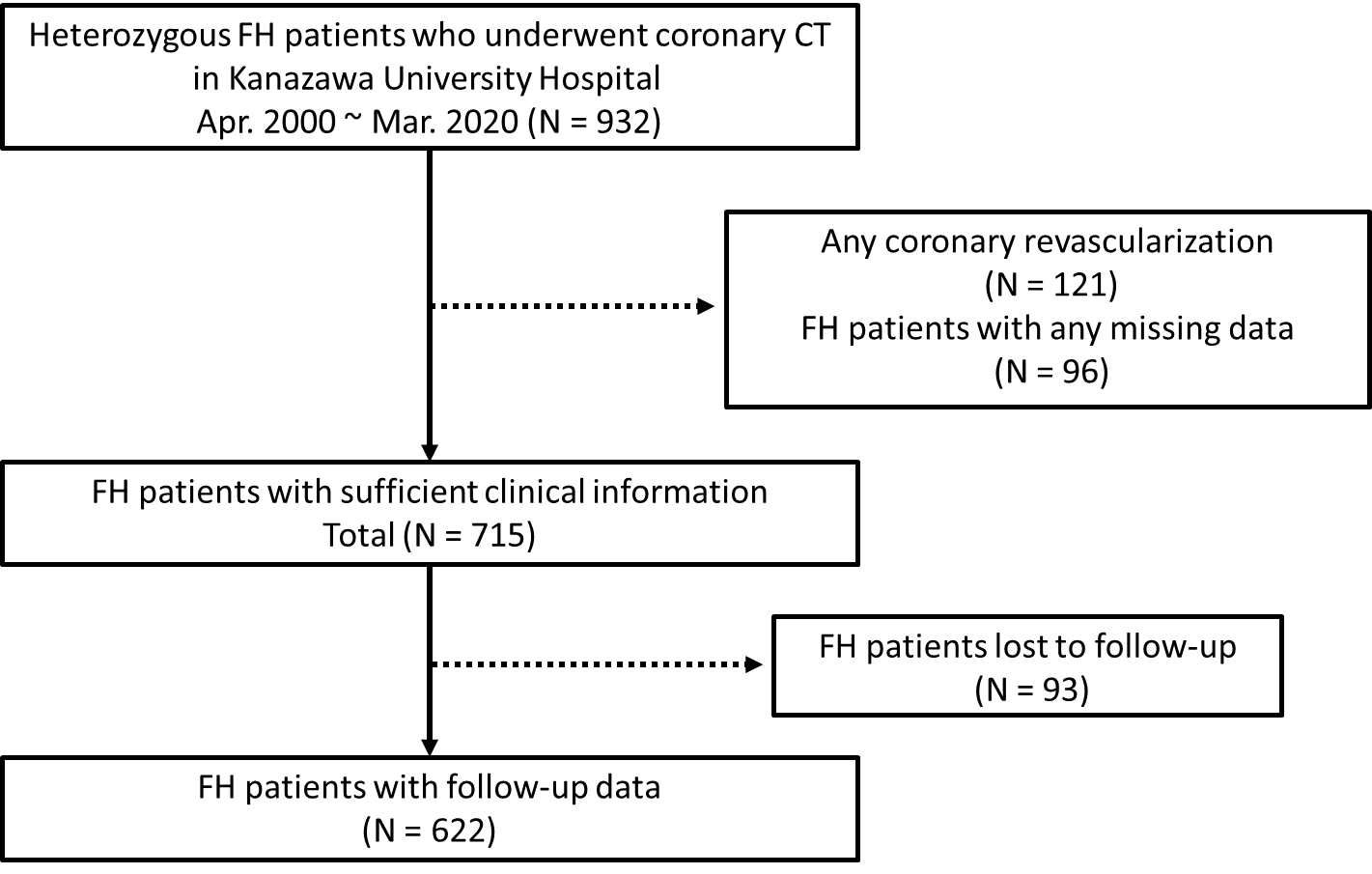
**

This flow diagram illustrates how the patients were selected and/or excluded from this study. FH, familial hypercholesterolemia; CT, computed tomography.

**Supplemental Figure 2. Prognosis according to LDL cholesterol treatment target**


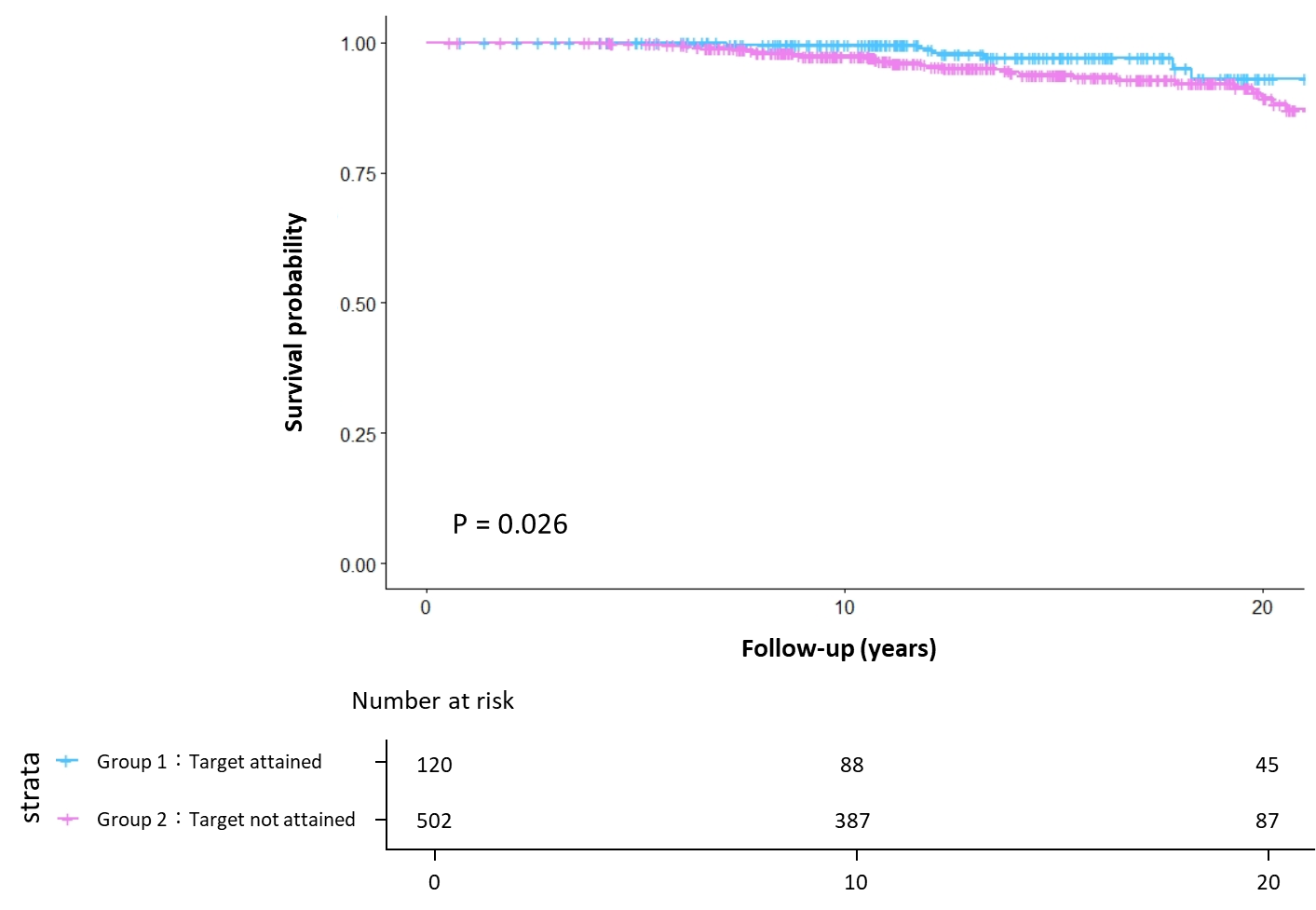


**Supplemental Table 1. Baseline characteristics according to CVD events.**

| Variables | All | CVD events | Without CVD events | *P*-value |
| --- | --- | --- | --- | --- |
|  | (N = 622) | (N = 132) | (N = 490) |  |
| Age (years) | 54 ± 13 | 61 ± 13 | 52 ± 13 | < 0.0001 |
| Male (%) | 306 (49.2%) | 82 (62.1%) | 224 (45.7%) | 0.0087 |
| Hypertension (%) | 200 (32.1%) | 89 (67.4%) | 111 (22.7%) | < 0.0001 |
| Diabetes (%) | 64 (10.3%) | 30 (22.7%) | 34 (6.9%) | < 0.0001 |
| Smoking (%) | 217 (34.9%) | 74 (56.1%) | 143 (29.2%) | < 0.0001 |
| Total cholesterol (mg/dL) | 318 [286–360] | 321 [287–389] | 316 [286–352] | 0.04 |
| Triglyceride (mg/dL) | 130 [91–176] | 133 [95–178] | 129 [89–173] | 0.173 |
| HDL cholesterol (mg/dL) | 46 [39–56] | 42 [34–51] | 48 [40–59] | < 0.0001 |
| LDL cholesterol (at baseline, mg/dL) | 229 [205–275] | 258 [216–310] | 225 [202–265] | < 0.0001 |
| LDL cholesterol (at follow-up mg/dL) | 108 [90–127] | 108 [89–129] | 107 [90–125] | 0.534 |
| LDL cholesterol year score (years × mg/dL) | 12692 [10085–15689] | 15733 [12894–19900] | 12006 [9768–14770] | < 0.0001 |
| FH pathogenic variants (%) | 425 (68.3%) | 115 (87.1%) | 310 (63.3%) | < 0.0001 |
| CAC score | 5 [0–50] | 104 [50–234] | 0 [0–12] | < 0.0001 |

FH, familial hypercholesterolemia; CAC, coronary artery calcium; CVD, cardiovascular event.

**Supplemental Table 2. Lipid-lowering therapies.**

| Lipid-lowering therapies | All | CVD events | Without CVD events |
| --- | --- | --- | --- |
|  | (N = 622) | (N = 132) | (N = 490) |
| Statins (%) | 610 (98.1%) | 131 (99.2%) | 479 (97.8%) |
| Ezetimibe (%) | 387 (62.2%) | 92 (69.7%) | 295 (60.2%) |
| Colestimide (%) | 135 (21.7%) | 28 (21.2%) | 107 (21.8%) |
| Probcol (%) | 1 (0.2%) | 1 (0.8%) | 0 (0.0%) |
| PCSK9 inhibitor (%) | 32 (5.1%) | 10 (7.6%) | 22 (4.5%) |
| LDL apheresis (%) | 1 (0.2%) | 1 (0.8%) | 0 (0.0%) |
| Fibrates (%) | 5 (0.8%) | 2 (1.5%) | 3 (0.6%) |
| n-3 PUFAs (%) | 9 (1.4%) | 3 (2.3%) | 6 (1.2%) |

PCSK9, proprotein convertase subtilisin/kexin type 9; PUFA, polyunsaturated fatty acid; CVD, cardiovascular disease; LDL, low-density lipoprotein.

**Supplemental Table 3. Identified mutations in FH genes.**

| Gene | Nucleotide change | Mutation Type | Effect on Protein | Number of Patients | ACMG |  |
| --- | --- | --- | --- | --- | --- | --- |
|  |  |  |  |  |  |  |
|  |  |  |  |  |  |  |
| *LDLR* | c.68-1G>C | Splice-cite | NA | 1 | PVS1/PM2/PM4/PP1/PP5 |  |
|  |  |  |  |  | Pathogenic |  |
| *LDLR* | c.130T>G | Missense | p.Trp44Gly | 3 | PM1/PM2/PP3/PP4 |  |
|  |  |  |  |  | Likely pathogenic |  |
| *LDLR* | c.137G>A | Missense | p.Cys46Tyr | 2 | PM1/PM2/PP3/PP5 |  |
|  |  |  |  |  | Likely Pathogenic |  |
| *LDLR* | c.191-2A>G | Splice-cite | NA | 2 | PVS1/PM2/PM4/PP1 |  |
|  |  |  |  |  | Pathogenic |  |
| *LDLR* | c.283T>G | Missense | p.Cys95Gly | 2 | PM1/PM2/PP3/PP4 |  |
|  |  |  |  |  | Likely pathogenic |  |
| *LDLR* | c.313+1G>A | Splice-cite | NA | 2 | PVS1/PM2/PM4 |  |
|  |  |  |  |  | Pathogenic |  |
| *LDLR* | c.361T>G | Missense | p.Cys121Gly | 2 | PM1/PM2/PP3/PP4/PP5 |  |
|  |  |  |  |  | Likely pathogenic |  |
| *LDLR* | c.378del | Frameshift | p.Phe126LeufsTer80 | 2 | PVS1/PM2/PM4/PP1 |  |
|  |  |  |  |  | Pathogenic |  |
| *LDLR* | c.389dupC | Frameshift | p.Asp131ArgfsTer49 | 5 | PVS1/PM1/PM2/PM4/PP5 |  |
|  |  |  |  |  | Pathogenic |  |
| *LDLR* | c.413C>G | Nonsense | p.Ser138Ter | 3 | PVS1/PM2/PM4/PP1/PP5 |  |
|  |  |  |  |  | Pathogenic |  |
| *LDLR* | c.418G>A | Missense | p.Glu140Lys | 4 | PM1/PM2/PP3/PP5 |  |
|  |  |  |  |  | Likely Pathogenic |  |
| *LDLR* | c.478T>C | Missense | p.Cys160Arg | 4 | PM1/PM2/PP3/PP5 |  |
|  |  |  |  |  | Likely Pathogenic |  |
| *LDLR* | c.489G>T | Missense | p.Gln163His | 4 | PM1/PM2/PP3/PP5 |  |
|  |  |  |  |  | Likely Pathogenic |  |
| *LDLR* | c.530C>T | Missense | p.Ser177Leu | 4 | PM1/PM2/PP3/PP5 |  |
|  |  |  |  |  | Likely Pathogenic |  |
| *LDLR* | c.532G>T | Missense | p.Asp178Tyr | 5 | PM1/PM2/PP3/PP5 |  |
|  |  |  |  |  | Likely Pathogenic |  |
| *LDLR* | c.611G>C | Missense | p.Cys204Ser | 6 | PM1/PM2/PP3/PP5 |  |
|  |  |  |  |  | Likely Pathogenic |  |
| *LDLR* | c.642G>C | Missense | p.Trp214Cys | 7 | PM1/PM2/PP3/PP5 |  |
|  |  |  |  |  | Likely Pathogenic |  |
| *LDLR* | c.682G>A | Missense | p.Glu228Gln | 5 | PM1/PM2/PP3/PP5 |  |
|  |  |  |  |  | Likely Pathogenic |  |
| *LDLR* | c.686_689del | Frameshift | p.Glu229AlafsTer35 | 3 | PVS1/PM2/PM4/PP1 |  |
|  |  |  |  |  | Pathogenic |  |
| *LDLR* | c.726G>C | Missense | p.Gln242His | 3 | PM1/PM2/PP3/PP4 |  |
|  |  |  |  |  | Likely Pathogenic |  |
| *LDLR* | c.796G>A | Missense | p.Asp266Asn | 2 | PM1/PM2/PP3/PP4 |  |
|  |  |  |  |  | Likely Pathogenic |  |
| *LDLR* | c.797A>G | Missense | p.Asp266Gly | 5 | PM1/PM2/PM5/PP1/PP3 |  |
|  |  |  |  |  | Likely Pathogenic |  |
| *LDLR* | c.829G>T | Nonsense | p.Glu277Ter | 2 | PVS1/PM2/PM4/PP1 |  |
|  |  |  |  |  | Pathogenic |  |
| *LDLR* | c.874delC | Frameshift | p.Leu292TrpfsTer78 | 3 | PVS1/PM2/PM4/PP1 |  |
|  |  |  |  |  | Pathogenic |  |
| *LDLR* | c.901G>T | Missense | p.Asp301Tyr | 4 | PM1/PM2/PP3/PP5 |  |
|  |  |  |  |  | Likely Pathogenic |  |
| *LDLR* | c.902A>T | Missense | p.Asp301Val | 4 | PM1/PM2/PP3/PP5 |  |
|  |  |  |  |  | Likely Pathogenic |  |
| *LDLR* | c.937T>G | Missense | p.Cys313Gly | 3 | PM1/PM2/PP3/PP4 |  |
|  |  |  |  |  | Likely Pathogenic |  |
| *LDLR* | c.939C>A | Missense | p.Cys313Ter | 2 | PVS1/PM2/PM4/PP5 |  |
|  |  |  |  |  | Pathogenic |  |
| *LDLR* | c.940+2T>C | Splice-cite | NA | 2 | PVS1/PM2/PM4/PP1 |  |
|  |  |  |  |  | Pathogenic |  |
| *LDLR* | c.967G>A | Missense | p.Gly323Ser | 4 | PM1/PM2/PP3/PP4 |  |
|  |  |  |  |  | Likely Pathogenic |  |
| *LDLR* | c.1007_1010delACGA | Frameshift | p.Tyr336CysfsTer33 | 2 | PVS1/PM2/PM4/PP5 |  |
|  |  |  |  |  | Pathogenic |  |
| *LDLR* | c.1012T>A | Missense | p.Cys338Ser | 2 | PM1/PM2/PP3/PP5 |  |
|  |  |  |  |  | Likely Pathogenic |  |
| *LDLR* | c.1056C>A | Nonsense | p.Cys352Ter | 2 | PVS1/PM2/PM4/PP5 |  |
|  |  |  |  |  | Pathogenic |  |
| *LDLR* | c.1062dupT | Frameshift | p.Ile355TyrfsTer3 | 2 | PVS1/PM2/PM4/PP5 |  |
|  |  |  |  |  | Pathogenic |  |
| *LDLR* | c.1067A>T | Missense | p.Asp356Val | 2 | PM1/PM2/PP3/PP4 |  |
|  |  |  |  |  | Likely Pathogenic |  |
| *LDLR* | c.1069G>T | Nonsense | p.Glu357Ter | 2 | PVS1/PM2/PM4 |  |
|  |  |  |  |  | Pathogenic |  |
| *LDLR* | c.1114_1115insC | Frameshift | p.Glu372AlafsTer9 | 3 | PVS1/PM2/PM4/PP5 |  |
|  |  |  |  |  | Pathogenic |  |
| *LDLR* | c.1187-2A>G | Splice-cite | NA | 3 | PVS1/PM2/PM4/PP5 |  |
|  |  |  |  |  | Pathogenic |  |
| *LDLR* | c.1207T>C | Missense | p.Phe403Leu | 2 | PM1/PM2/PP3/PP5 |  |
|  |  |  |  |  | Likely Pathogenic |  |
| *LDLR* | c.1245_1249dupCCGGA | Frameshift | p.Ser417ThrfsTer12 | 4 | PVS1/PM2/PM4/PP4 |  |
|  |  |  |  |  | Pathogenic |  |
| *LDLR* | c.1246C>T | Missense | p.Arg416Trp | 4 | PM1/PM2/PP3/PP4 |  |
|  |  |  |  |  | Likely Pathogenic |  |
| *LDLR* | c.1252G>A | Missense | p.Glu418Lys | 4 | PM1/PM2/PP3/PP4 |  |
|  |  |  |  |  | Likely Pathogenic |  |
| *LDLR* | c.1285G>A | Missense | p.Val429Leu | 4 | PM1/PM2/PP3/PP4 |  |
|  |  |  |  |  | Likely Pathogenic |  |
| *LDLR* | c.1297G>C | Missense | p.Asp433His | 4 | PM1/PM2/PP3/PP5 |  |
|  |  |  |  |  | Likely Pathogenic |  |
| *LDLR* | c.1328G>C | Missense | p.Trp443Ser | 4 | PM1/PM2/PP3/PP4 |  |
|  |  |  |  |  | Likely Pathogenic |  |
| *LDLR* | c.1339T>C | Missense | p.Ser447Pro | 3 | PM1/PM2/PP3/PP5 |  |
|  |  |  |  |  | Likely Pathogenic |  |
| *LDLR* | c.1340C>G | Missense | p.Ser447Cys | 3 | PM1/PM2/PP3/PP5 |  |
|  |  |  |  |  | Likely Pathogenic |  |
| *LDLR* | c.1432G>A | Missense | p.Gly478Arg | 2 | PM1/PM2/PP3/PP4 |  |
|  |  |  |  |  | Likely Pathogenic |  |
| *LDLR* | c.1466A>G | Missense | p.Tyr489Cys | 3 | PM1/PM2/PP3/PP5 |  |
|  |  |  |  |  | Likely Pathogenic |  |
| *LDLR* | c.1474G>A | Missense | p.Asp492Asn | 4 | PM1/PM2/PP3/PP4 |  |
|  |  |  |  |  | Likely Pathogenic |  |
| *LDLR* | c.1502C>T | Missense | p.Ala501Val | 5 | PM1/PM2/PP3/PP4 |  |
|  |  |  |  |  | Likely Pathogenic |  |
| *LDLR* | c.1567G>A | Missense | p.Val523Met | 3 | PM1/PM2/PP3/PP5 |  |
|  |  |  |  |  | Likely Pathogenic |  |
| *LDLR* | c.1586+1G>A | Splice-cite | NA | 2 | PVS1/PM2/PM4/PP4 |  |
|  |  |  |  |  | Pathogenic |  |
| *LDLR* | c.1652_1662delACATCTACTCG | Frameshift | p.Asp551AlafsTer4 | 4 | PVS1/PM2/PM4/PP4 |  |
|  |  |  |  |  | Pathogenic |  |
| *LDLR* | c.1702C>G | Missense | p.Leu568Val | 6 | PM1/PM2/PP3/PP5 |  |
|  |  |  |  |  | Likely Pathogenic |  |
| *LDLR* | c.1705+1G>C | Splice-cite | NA | 4 | PVS1/PM2/PM4/PP4 |  |
|  |  |  |  |  | Pathogenic |  |
| *LDLR* | c.1706A>G | Missense | p.Asp569Gly | 2 | PM1/PM2/PP3/PP4 |  |
|  |  |  |  |  | Likely Pathogenic |  |
| *LDLR* | c.1727A>G | Missense | p.Tyr576Cys | 5 | PM1/PM2/PP3/PP4 |  |
|  |  |  |  |  | Likely Pathogenic |  |
| *LDLR* | c.1731G>T | Missense | p.Trp577Cys | 5 | PM1/PM2/PP3/PP4 |  |
|  |  |  |  |  | Likely Pathogenic |  |
| *LDLR* | c.1778dupG | Frameshift | p.Asn594GlnfsTer9 | 5 | PVS1/PM2/PM4/PP4 |  |
|  |  |  |  |  | Pathogenic |  |
| *LDLR* | c.1783C>T | Missense | p.Arg595Trp | 4 | PM1/PM2/PP3/PP4 |  |
|  |  |  |  |  | Likely Pathogenic |  |
| *LDLR* | c.1845+2T>C | Splice-cite | NA | 4 | PVS1/PM2/PM4/PP4 |  |
|  |  |  |  |  | Pathogenic |  |
| *LDLR* | c.1859G>C | Missense | p.Trp620Ser | 4 | PM1/PM2/PP3/PP4 |  |
|  |  |  |  |  | Likely Pathogenic |  |
| *LDLR* | c.1868T>A | Missense | p.Ile623Asn | 3 | PM1/PM2/PP3/PP4 |  |
|  |  |  |  |  | Likely Pathogenic |  |
| *LDLR* | c.1897C>T | Missense | p.Arg633Cys | 4 | PM1/PM2/PP3/PP4 |  |
|  |  |  |  |  | Likely Pathogenic |  |
| *LDLR* | c.1925T>C | Missense | p.Leu642Ser | 3 | PM1/PM2/PP3/PP4/PP5 |  |
|  |  |  |  |  | Likely Pathogenic |  |
| *LDLR* | c.1998G>C | Missense | p.Trp666Cys | 4 | PM1/PM2/PP3/PP4 |  |
|  |  |  |  |  | Likely Pathogenic |  |
| *LDLR* | c.2050G>T | Missense | Ala684Ser | 4 | PM1/PM2/PP3/PP4 |  |
|  |  |  |  |  | Likely Pathogenic |  |
| *LDLR* | c.2054C>T | Missense | p.Pro685Leu | 24 | PM1/PM2/PP3/PP4/PP5 |  |
|  |  |  |  |  | Likely Pathogenic |  |
| *LDLR* | c.2096C>T | Missense | p.Pro699Leu | 4 | PM1/PM2/PP3/PP4 |  |
|  |  |  |  |  | Likely Pathogenic |  |
| *LDLR* | c.2389G>A | Missense | p.Val797Met | 6 | PM1/PM2/PP3/PP4 |  |
|  |  |  |  |  | Likely Pathogenic |  |
| *LDLR* | c.2390-4_2393delACAGTGCT | Splice-cite | NA | 2 | PVS1/PM2/PM4/PP4 |  |
|  |  |  |  |  | Pathogenic |  |
| *LDLR* | c.2416delG | Frameshift | p.Val806SerfsTer123 | 3 | PVS1/PM2/PM4/PP4 |  |
|  |  |  |  |  | Pathogenic |  |
| *LDLR* | c.2416dupG | Frameshift | p.Val806GlyfsTer11 | 4 | PVS1/PM2/PM4/PP4 |  |
|  |  |  |  |  | Pathogenic |  |
| *LDLR* | c.2431A>T | Nonsense | p.Lys811Ter | 112 | PVS1/PM2/PM4/PP4 |  |
|  |  |  |  |  | Pathogenic |  |
| *LDLR* | c.2500G>A | Missense | p.Asp834Asn | 2 | PVS1/PM2/PM4/PP4 |  |
|  |  |  |  |  | Pathogenic |  |
| *LDLR* | c.2579C>T | Missense | p.Ala860Val | 2 | PVS1/PM2/PM4/PP4 |  |
|  |  |  |  |  | Pathogenic |  |
| *LDLR* | c.313-?_2311+?del | Large deletion | Truncated protein | 2 | PVS1/PM2/PM4/PP4 |  |
|  |  |  |  |  | Pathogenic |  |
| *LDLR* | c.1186-?_1587+?dup | Large duplication | Truncated protein | 2 | PVS1/PM2/PM4/PP4 |  |
|  |  |  |  |  | Pathogenic |  |
| *LDLR* | c.1845-?_2141+?del | Large deletion | Truncated protein | 1 | PVS1/PM2/PM4/PP4 |  |
|  |  |  |  |  | Pathogenic |  |
| *LDLR* | c.2141-?_2311+?del | Large deletion | Truncated protein | 3 | PVS1/PM2/PM4/PP4 |  |
|  |  |  |  |  | Pathogenic |  |
| *PCSK9* | c.94G>A | Missense | p.Glu32Lys | 28 | PS1/PS3/PP3/PP4/PP5 |  |
|  |  |  |  |  | Pathogenic |  |

ACMG, American College of Medical Genetics; *LDLR*, low-density lipoprotein receptor; *PCSK9*, proprotein convertase subtilisin/kexin type 9; FH, familial hypercholesterolemia.

**Supplemental Table 4. Type of CVD events.**

| Type of CVD events | All (N = 622) |  |
| --- | --- | --- |
|  |  |  |
| CVD-associated death | 20 (3.2 %) |  |
| Myocardial infarction | 9 (1.4 %) |  |
| Unstable angina | 18 (2.9 %) |  |
| Staged revascularization | 85 (13.7 %) |  |
| Total | 132 (21.2 %) |  |

CVD, cardiovascular disease.

**Supplemental Table 5. Factors associated with CVD events (Fine-Gray regression model).**

| Variable | HR | 95% CI | *P*-value |
| --- | --- | --- | --- |
| Age (per year) | 1.06 | 1.04–1.08 | < 0.0001 |
| Male (yes vs. no) | 1.48 | 1.04–2.10 | 0.014 |
| Hypertension (yes vs. no) | 2.32 | 1.60–3.24 | < 0.0001 |
| Diabetes (yes vs. no) | 2.08 | 1.18–2.96 | 0.0002 |
| Smoking (yes vs. no) | 2.86 | 1.84–3.88 | 0.0001 |
| LDL cholesterol (per 10 mg/dL) | 1.01 | 1.00–1.02 | 0.021 |
| Pathogenic variants (*vs.* without variants) | 2.88 | 1.84–3.92 | < 0.0001 |
| Log (CAC + 1) | 3.14 | 1.56–4.76 | < 0.0001 |

HR, hazard ratio; CI, confidence interval; CAC, coronary artery calcium; CVD, cardiovascular disease.
